# Advancing palliative medicine training in adolescent and young adult care: a needs assessment study

**DOI:** 10.1186/s12909-026-09774-8

**Published:** 2026-06-22

**Authors:** Abdulaziz Bakhsh, Amirrtha Srikanthan, Sarah Cleyn, Stephanie C.M. Veldhuijzen van Zanten, Perri Tutelman, Anne Marie Krueger-Naug, Henrique Parsons, Julie Lapenskie, Sydney Ruller, Samantha Halman, James Downar, Mohamed Abdelaal

**Affiliations:** 1 Division of Palliative Care, Department of Medicine, University of Ottawa, 501 Smyth Rd, Ottawa, ON K1H 8L6 Canada; 2 Division of Medical Oncology, Department of Medicine, University of Ottawa, Ottawa, ON Canada; 3 The Ottawa Hospital, Ottawa, ON Canada; 4 Pediatric Palliative Care, Children’s Hospital of Eastern Ontario, Ottawa, ON Canada; 5 Department of Psychology, University of Calgary, Calgary, AB Canada; 6 Division of Palliative Medicine, Department of Medicine, Dalhousie University, Halifax, NS Canada; 7 Ottawa Hospital Research Institute, Ottawa, ON Canada; 8 Bruyère Health Research Institute, Ottawa, ON Canada; 9 Department of Medicine, The Ottawa Hospital, Ottawa, ON Canada; 10 Department of Medicine, University of Ottawa, Ottawa, ON Canada

**Keywords:** Adolescents, Young adults, Palliative care, Medical education, Needs assessment

## Abstract

**Background:**

Adolescents and young adults (AYAs), defined as individuals aged 15–39 years, face unique medical, psychosocial, and existential challenges when living with life-limiting illness. Despite evidence supporting early palliative care integration, Canadian adult palliative care curricula lack formalized AYA-specific competencies. Healthcare providers consistently report insufficient preparation in this area.

**Methods:**

A descriptive cross-sectional needs assessment survey was administered nationally to physicians who completed Canadian adult palliative care residency training between 2019 and 2024. A multidisciplinary steering committee of experts in AYA palliative care and medical education, alongside a patient partner, guided the development of the bilingual 25-item questionnaire guided by an established framework for medical education survey design. The questionnaire assessed clinical exposure, self-rated knowledge across 20 competency areas, comfort levels across 21 competency areas, training needs and barriers, and preferred learning formats. Responses were anonymous. Descriptive statistics were used for analysis.

**Results:**

Of 71 responses received, 55 met eligibility criteria for analysis (response rate 35%, eligibility rate 77%). Only 31% of participants received AYA-specific training during residency. Most participants (82%) reported low comfort when caring for the youngest AYA age group (15–18 years). The greatest knowledge gaps were in pediatric-to-adult care transitions (69%), sexual health and fertility concerns (62%), and social support navigation (39%). Top training priorities were AYA developmental and psychosocial factors (75%), grief and bereavement support (65%), and care transitions (60%). The most common training barriers were limited clinical exposure (78%), lack of mentors (65%), and absence of AYA-specific programs (61%).

**Conclusions:**

Our findings revealed substantial gaps in training, knowledge, and comfort levels in providing AYA palliative care among recent graduates from the adult palliative care programs in Canada. This may represent an initial step towards the development of a competency-based AYA-focused residency training curriculum.

**Supplementary Information:**

The online version contains supplementary material available at https://doi.org/10.1186/s12909-026-09774-8.

## Introduction

Adolescents and young adults (AYAs), typically defined as individuals aged 15–39 years, represent a unique and underserved population in palliative care [1]. When facing life-limiting illnesses, AYAs experience a unique confluence of medical, psychosocial, and developmental challenges distinct from those of younger children or older adults [2–4]. Evidence demonstrates that early integration of palliative care can improve symptom control, enhance communication about treatment goals, and provide essential psychosocial support for patients and families facing life-limiting illnesses [2, 4]. However, there are several barriers to palliative care involvement with AYAs, such as the stigma around palliative care and its association with end of life [4, 5]. Additionally, healthcare providers caring for AYA patients often report insufficient preparation and lack of medical training in managing the end of life period for this population [6]. Many healthcare professionals may lack the skills, competence, and confidence to meet the needs of AYAs [7].

Palliative care training programs emphasize holistic care [2], yet there are substantial gaps in AYA-specific education [8]. In Canada, postgraduate medical education has shifted toward Competence By Design (CBD), with the CanMEDS framework providing structure for developing measurable competencies aligned with national training standards [9]. While comprehensive adult and pediatric palliative care curricula exist in Canada, formalized AYA-specific palliative care competencies remain underdeveloped.

Very little is known about the knowledge gaps and training needs of physicians providing palliative care to AYAs. To our knowledge, no previous studies have systematically assessed the learning experiences, perceived preparedness, or identified training needs of graduates from Canadian adult palliative care residency programs regarding AYA care.

The aim of our study was to conduct a needs assessment of recent graduates from the Canadian adult palliative care programs. Specifically, the study sought to evaluate graduates’ clinical exposure and comfort levels with AYA patients, identify training gaps in symptom management and communication skills, and determine preferences for curriculum improvement. Our findings provide the first step towards developing formal AYA-specific competencies and educational frameworks for adult palliative care training in Canada for both the Family Medicine and the Royal College training routes.

## Methods

### Study design

This is a descriptive, cross-sectional needs assessment survey of recent graduates from Canadian adult palliative care residency programs.

### Survey development

Prior to survey development, we created a multidisciplinary steering committee, composed of experts in AYA palliative care (*n* = 4) and medical education (*n* = 4), along with a patient partner. The steering committee members represented Eastern, Central, and Western Canadian provinces (Nova Scotia, Ontario, and Alberta) and a range of professional roles, including adult and pediatric palliative care physicians, pediatric and adult medical oncologists, an internist, a psychologist, and an advanced practice nurse. The inclusion of a patient partner ensured incorporation of patient perspectives regarding the required level of knowledge.

Following this, we completed a literature search to gather what is known about the existing knowledge gaps and training needs of healthcare professionals caring for AYA patients, and to guide the development of this study. We conducted searches of electronic databases (PubMed, Web of Science) and Google for published English literature, guidelines, and frameworks. There were no limitations on year of publication or country of origin.

Informed by our literature review [6–8, 10] and steering committee expertise, core competencies in AYA palliative care were identified including communication skills, symptom management, psychosocial care, fertility and sexuality, grief and bereavement, and end of life care. Based on these competencies, a questionnaire was developed to assess the participants’ knowledge and comfort levels in providing AYA palliative care, and any perceived training gaps (Supplementary Material 1). The survey included 25 questions in six sections: (i) demographic and training background; (ii) exposure and experience with AYA patients; (iii) educational needs and gaps; (iv) training barriers; (v) preferred learning methods; and (vi) final comments. The survey was officially translated by the Ottawa Hospital Research Institute, and was available in both English and French. The survey was administered electronically using LimeSurvey.

Knowledge and comfort were assessed using 5-point Likert scales ranging from “not at all knowledgeable/comfortable” to “extremely knowledgeable/comfortable.” Additionally, the survey included open-ended questions and dedicated space for participants to self-identify any training gaps.

We used Artino et al.’s framework for medical education survey design as a methodological guide for survey development and validation [11]. After the survey was developed, collective feedback was obtained from the steering committee members followed by an iterative question by question review of the survey items with the committee members to assess items wording, structure of the survey sections, and relevance to practice. Once finalized, the survey was pilot tested on a small group of palliative care physicians (*n* = 4) for clarity, format and flow. This resulted in changes to the survey format, before it was distributed nationally.

### Participants

Eligible participants were physicians who completed adult palliative care residency training between 2019 and 2024 from any Royal College of Physicians and Surgeons of Canada (RCPSC) Adult Palliative Medicine program or College of Family Physicians of Canada (CFPC) Enhanced Skills program in palliative care. Our inclusion criteria aimed to capture recent knowledge and clinical experience to reflect the training preparedness among the graduates.

### National distribution and data collection

Invitation emails were sent to physicians by the Canadian Society of Palliative Medicine (CSPM) and the Pan-Canadian Palliative Care Research Collaborative (PCPCRC). Two follow-up reminder emails, three weeks apart, were sent to potential participants. The survey remained active for eight weeks to allow enough time for participation.

Reviewing the Canadian Resident Matching Service (CaRMS) reports [12], we expected that 155 physicians completed Canadian palliative care programs between 2019 and 2024. We anticipated a 20% response rate to our survey. At the end of the survey, participants were offered an electronic gift card as an incentive for their participation.

### Ethical considerations

The study was approved by the Ottawa Health Science Network Research Ethics Board (Protocol#: 20250290–01 H), and was conducted in accordance with the ethical principles of the Declaration of Helsinki. Participants were provided detailed information regarding the study purpose, voluntary participation, anonymity, and their right to withdraw. Implied informed consent was obtained through survey completion, consistent with standard procedures for low-risk research. All data were stored securely on password-protected servers in accordance with institutional policies and Canadian privacy legislation.

### Data analysis

Participants with incomplete demographic data were excluded to ensure alignment with our eligibility criteria. For included participants, responses were collected from subsequent survey sections regardless of their completion rate. As specified in the consent process, participants were informed that all recorded responses would be included in the analysis even if they withdrew prior to survey completion.

Descriptive statistics were used to analyze quantitative data. Likert-scale responses were analyzed as proportions within each response category. Missing data were handled using available-case analysis, with percentages calculated based on the number of respondents to each individual question. All analyses were conducted using LimeSurvey^®^ and verified independently by two members of the study team.

## Results

### Demographic characteristics

Of 71 survey responses received, 16 were excluded due to incomplete demographic data, yielding 55 eligible participants for analysis (response rate 35%, eligibility rate 77%). The majority of participants were 30–35 years old (47%, *n* = 26), female (66%, *n* = 36), and graduated from an Enhanced Skills program accredited by the College of Family Physicians of Canada (75%, *n* = 41) (Table 1). The largest proportion of participants completed their palliative care training in 2024 (27%, *n* = 15).

### AYA training and comfort levels

Only one-third of the participants reported receiving any AYA-specific training during residency (31%, *n* = 17). Among the 17 participants who received AYA-specific training, adult palliative care clinical rotations (82%, *n* = 14) and pediatric palliative care rotations (71%, *n* = 12) were the most common training modalities.

**Table 1:** Demographics

Characteristic (*n* = 55)	*n* (%)
Age	
Less than 30 years	5 (9.1)
30–35 years	26 (47.3)
36–40 years	16 (29.1)
41–45 years	4 (7.3)
46–50 years	4 (7.3)
Sex	
Female	36 (65.5)
Male	19 (34.5)
Training Pathway	
CFPC† Enhanced Skills in Palliative Care	41 (74.5)
RCPSC‡ Palliative Medicine	6 (10.9)
Other^*^	8 (14.5)
Year of Training Completion	
2019	9 (16.4)
2020	5 (9.1)
2021	10 (18.2)
2022	7 (12.7)
2023	9 (16.4)
2024	15 (27.3)
Province of Training	
Ontario	33 (60.0)
Alberta	6 (10.9)
British Columbia	4 (7.3)
Manitoba	4 (7.3)
Quebec	4 (7.3)
Nova Scotia	3 (5.5)
Newfoundland and Labrador	1 (1.8)
Practice Setting**	
Academic hospital	39 (70.9)
Home-based care	25 (45.5)
Outpatient clinic	19 (34.5)
Community hospital	19 (34.5)
Hospice	11 (20.0)
Received AYA-Specific Training**	
No	38 (69.1)
Yes	17 (30.9)
· Lectures or classroom-based learning	10 (18.2)
· Workshops or simulations	1 (1.8)
· Adult palliative care clinical rotations	14 (25.5)
· Pediatric palliative care clinical rotations	12 (21.8)
· Online modules or e-learning	3 (5.5)

† *CFPC*  College of Family Physicians of Canada

‡ *RCPSC*  Royal College of Physicians and Surgeons of Canada

*Other: University of Toronto clinical fellowship, University of British Columbia category 2 enhanced skills, Family medicine 12 weeks enhanced skills in palliative care

** Respondents could select multiple answers

### Comfort levels by patient age group

**Fig. 1:**
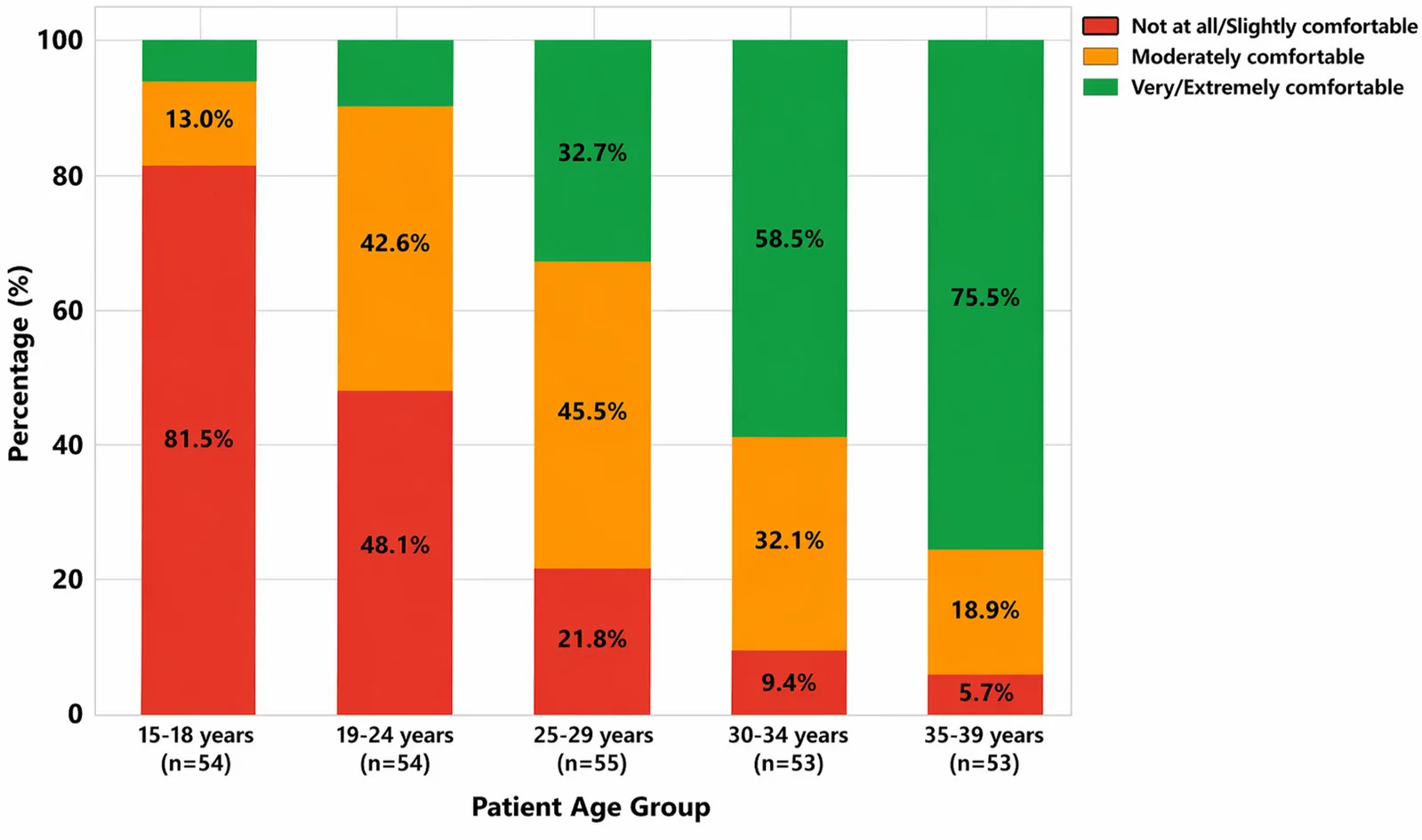
Comfort levels providing palliative care by patient age group

Most participants (82%, *n* = 44) reported low comfort levels (not at all or slightly comfortable) when providing care to the youngest AYA age group (15–18 years old); of these, 23% (*n* = 10) had completed a pediatric palliative care rotation during residency. In contrast, 76% (*n* = 40) reported being (very/extremely comfortable) with AYAs aged 35–39 years (Fig. 1).

### Palliative care training gaps

**Fig. 2:**
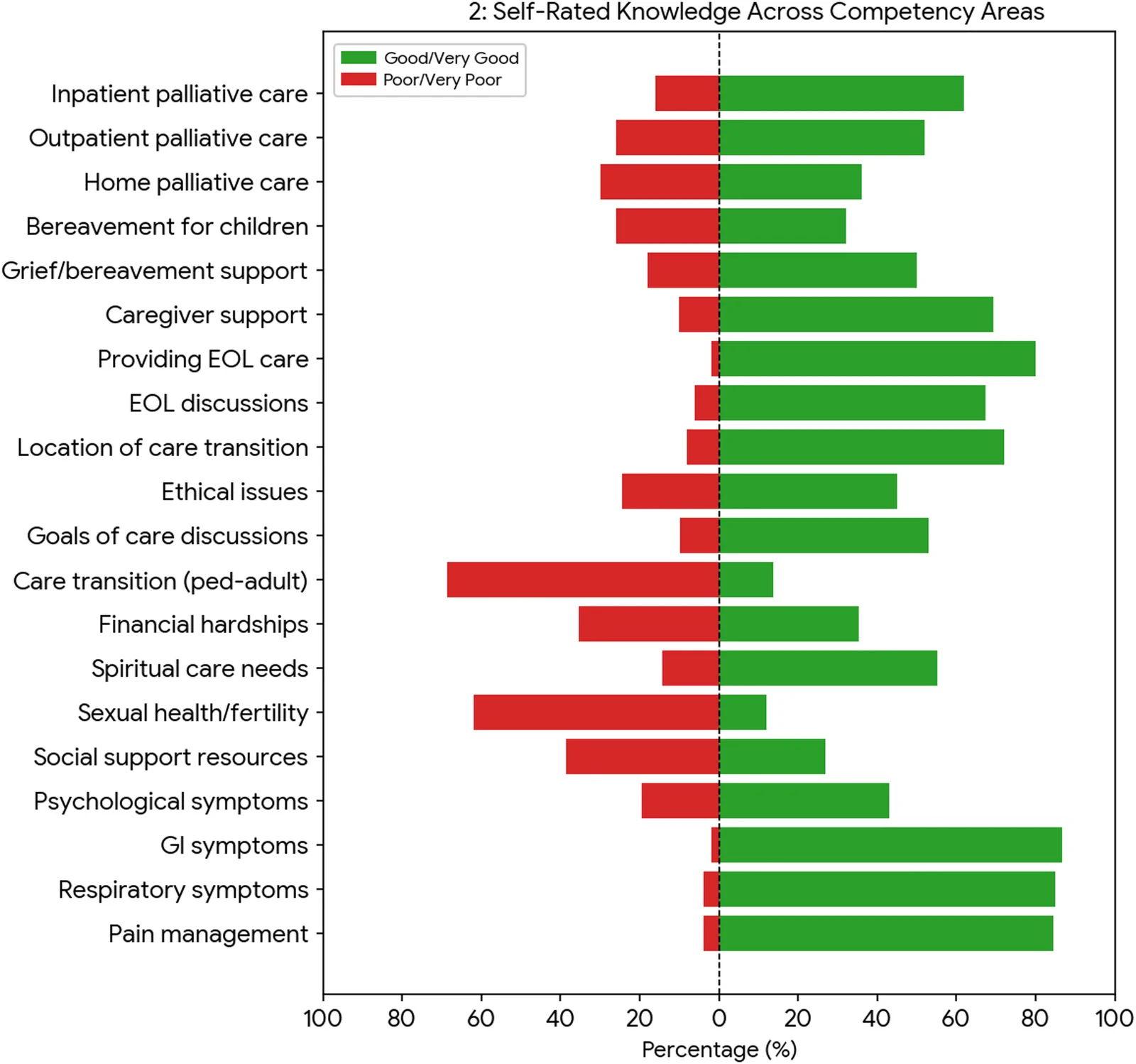
Self-rated knowledge across competency areas

Participants rated their knowledge across 20 core competency areas (Fig. 2). Most participants reported good/very good knowledge levels in gastrointestinal symptom management (87%, *n* = 46), respiratory symptom management (85%, *n* = 45), and pain management (85%, *n* = 44). Conversely, poor/very poor knowledge levels were reported in the process of care transition from pediatric to adult care (69%, *n* = 35), exploring sexual health and fertility concerns (62%, *n* = 31), and navigating social support resources (39%, *n* = 20).

**Fig. 3:**
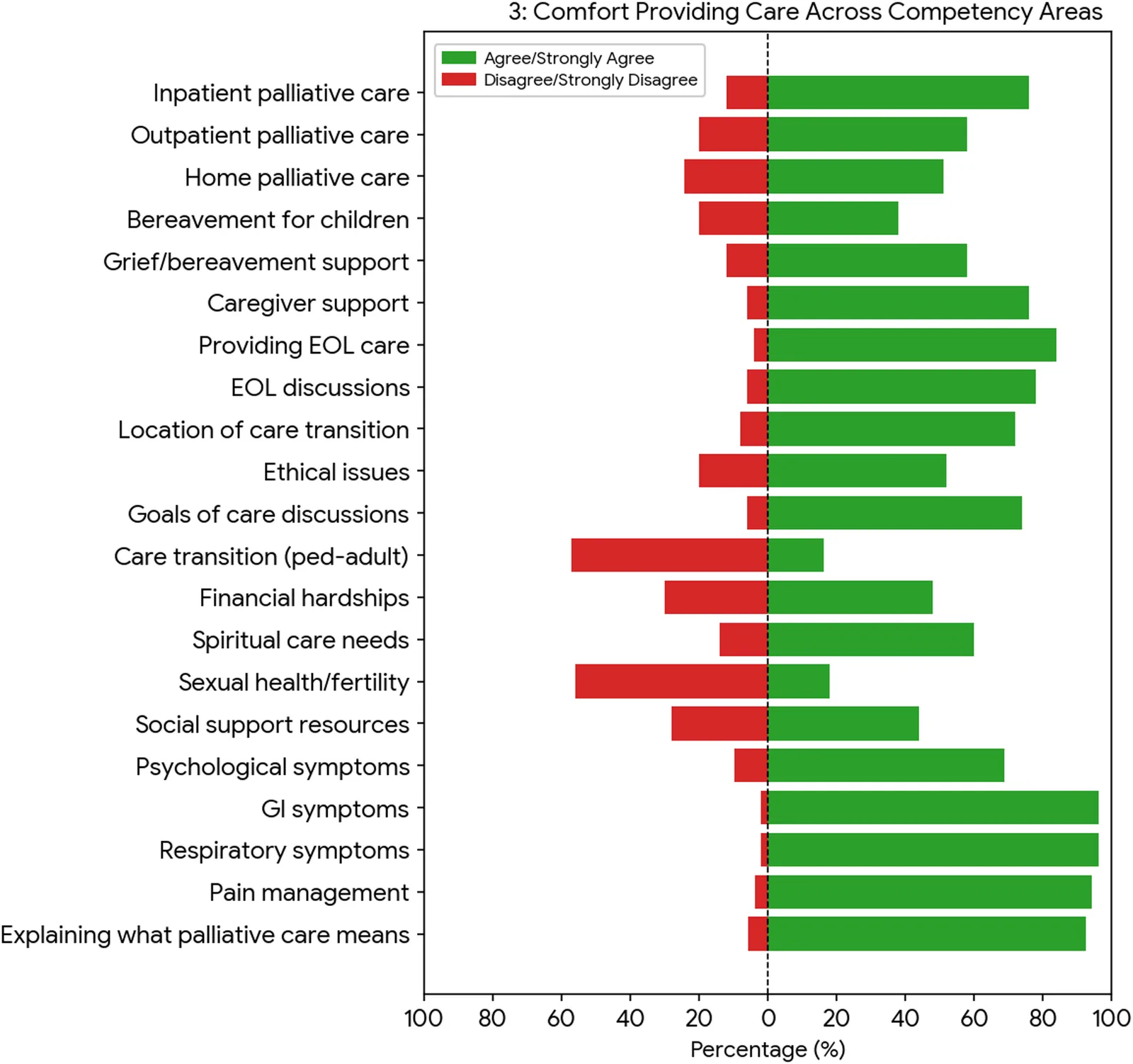
Comfort levels across competency areas

Comfort levels were also assessed across 21 competency areas (Fig. 3). High comfort levels (agree/strongly agree) were identified for managing gastrointestinal symptoms (96%, *n* = 49) and respiratory symptoms (96%, *n* = 49). Low comfort levels (disagree/strongly disagree) were reported for the process of care transition from pediatric to adult care (57%, *n* = 28) and exploring sexual health concerns (56%, *n* = 28). Full results of knowledge and comfort levels are available in Supplementary Material 2.

Additionally, we examined the challenges reported by participants in providing AYA palliative care during their residency training (Table 2). Participants identified the following areas as “very/extremely challenging”: emotional burden of working with younger patients (46%, *n* = 23), lack of clinical exposure to AYA patients during training (38%, *n* = 19), and lack of AYA-specific resources at the training site (34%, *n* = 17).

**Table 2:** Level of challenges during residency training

Area	Challenge level, *n* (%)^*^		
	High	Moderate	Low
Limited Knowledge of AYA-specific needs	12 (24)	22 (44)	16 (32)
Lack of clinical exposure to AYA patients during training	19 (38)	15 (30)	16 (32)
Time constraints during patient care	3 (6)	16 (32)	31 (62)
Difficulty in addressing psychosocial concerns	13 (27.1)	21 (43.8)	14 (29.2)
Emotional burden of working with younger patients	23 (46)	17 (34)	10 (20)
Lack of AYA-specific resources at the training site	17 (34)	19 (38)	14 (28)
Limited interdisciplinary collaboration for AYA care	11 (22)	15 (30)	14 (48)

^*^Percentages are based on the total number of eligible answers in each question. Item-specific denominator ranging from *n* = 48 to *n* = 50 due to missing data

### Perceived training needs

Participants identified the need for formal training across multiple domains (Table 3). The following training topics were identified as high priority: understanding developmental, psychological, and social factors unique to AYAs (75%, *n* = 38); grief and bereavement support for patients and families (65%, *n* = 33); process of care transition from pediatric to adult care (60%, *n* = 30); engaging caregivers in shared decision-making (58%, *n* = 29); and communicating bad news and end of life discussions (57%, *n* = 29).

**Table 3:** Perceived training needs for AYA palliative care

Training Area	Priority level, *n* (%)^*^		
	High	Moderate	Low
Symptom management	16 (30.8)	10 (19.2)	26 (50)
Pharmacology of medications used for AYA care	16 (31.4)	15 (29.4)	20 (39.2)
Communicating bad news and end of life discussions with AYAs	29 (56.9)	13 (25.5)	9 (17.6)
Grief and bereavement support for patients and families	33 (64.7)	14 (27.5)	4 (7.8)
Understanding developmental, psychological, and social factors unique to AYAs	38 (74.5)	12 (23.5)	1 (2)
Process of care transition from pediatric to adult care	30 (60)	17 (34)	3 (6)
Caregiver engagement in shared decision-making	29 (58)	13 (26)	8 (16)
Outpatient palliative care	23 (46)	15 (30)	12 (24)
Home-based palliative care	22 (44)	16 (32)	12 (24)
Inpatient palliative care	22 (44)	14 (28)	14 (28)

^*^ Percentages are based on the total number of eligible answers in each question. Item-specific denominator ranging from *n* = 50 to *n* = 52 due to missing data

### Training barriers and preferred learning formats

Limited clinical exposure to AYA patients was the most common learning barrier identified by participants (78%, *n* = 40), followed by lack of mentors or role models (65%, *n* = 33) and lack of available AYA-specific training programs (61%, *n* = 31) (Table 4).

**Table 4:** Training barriers

Identified training barriers (*n* = 51)	*n* (%)
Insufficient allocated time within the residency training	25 (49)
Limited clinical exposure to AYA patients	40 (78.4)
Lack of mentors or role models experienced in AYA care	33 (64.7)
Institutional focus on other patient populations	23 (45.1)
Lack of available AYA-specific training programs	31 (60.8)

Finally, we examined the participants’ preferred learning formats (Table 5). Clinical exposure through structured rotations, mentorship, and case-based learning were the preferred learning formats for 88% (*n* = 43), 73% (*n* = 37), and 67% (*n* = 32) of participants, respectively.

**Table 5:** Preferred learning formats

Learning format	Usefulness level, *n* (%)^*^		
	High	Moderate	Low
Online e-learning modules	9 (18)	15 (30)	26 (52)
In-person workshops	26 (52)	15 (30)	9 (18)
Mentorship	37 (72.5)	11 (21.6)	3 (5.9)
Self-learning through reading literature	5 (9.8)	14 (27.5)	32 (62.7)
Group discussions or peer support groups	20 (39.2)	19 (37.3)	12 (23.5)
Case-based learning	32 (66.7)	10 (20.8)	6 (12.5)
Clinical exposure through structured rotations	43 (87.8)	4 (8.2)	2 (4.1)

^*^ Percentages are based on the total number of eligible answers in each question. Item-specific denominator ranging from *n* = 48 to *n* = 51 due to missing data

## Discussion

To our knowledge, this is the first study to describe the self-identified educational needs of recent graduates from Canadian adult palliative care residency programs in providing AYA care. Our findings reveal substantial self-perceived gaps in training, knowledge, and comfort, particularly in caring for the youngest AYA age groups and in managing complex psychosocial and transitional care domains. These training gaps may influence the development of standardized, competency-based AYA palliative care curricula, building on the successful CBD implementation in Canada [6, 10, 13].

In general, participants reported varying degrees of comfort when providing care across the AYA age spectrum, with the vast majority reporting the lowest comfort levels caring for patients aged 15–18 years. Notably, this discomfort persisted even among participants who had completed a pediatric palliative care rotation. These findings suggest that pediatric rotations alone may be insufficient to prepare trainees for the unique needs of adolescents. These findings align with the existing literature that describes the AYA population as a vulnerable group, falling into a gap between pediatric and adult healthcare systems, often treated in disparate settings with different protocols [6, 14]. This pattern mirrors international findings, where palliative care was identified as a prominent educational need among Australian AYA providers [7]. The observed discomfort may result from insufficient clinical exposure to this age group, which may potentially contribute to the high rates of intensive end of life care documented among AYAs [15, 16].

The largest gaps in self-reported knowledge were observed in psychosocial and transitional care domains. Many graduates reported low knowledge and comfort levels in the process of transition from pediatric to adult care, and in addressing sexual health and fertility concerns of AYAs. Potential barriers to discussing fertility preservation may include limited knowledge and discomfort with the topic [17]. In addition, it may reflect a broader discomfort among healthcare professionals when addressing sexuality and fertility, particularly in the context of life-limiting illness, where these issues may be mistakenly viewed as less relevant [18, 19]. Similarly, navigating the transition from pediatric to adult care systems is a well-documented barrier to seamless care, with systemic issues including poor communication between pediatric and adult providers and a lack of training in managing the process [14, 20]. These gaps can directly affect patients, resulting in unmet needs and suboptimal care coordination.

Our study highlights some of the personal and systemic challenges that trainees face when caring for AYAs. The emotional burden of caring for younger patients was identified as the most significant challenge during residency training. This resonates with research describing the profound emotional impact and potential for burnout among professionals caring for this population, with documented impacts on quality of care, patient safety, and provider wellbeing [6, 21]. This emotional toll is compounded by systemic challenges, with limited clinical exposure to AYA patients being the most frequently reported barrier to training, followed by lack of experienced mentors and absence of dedicated AYA-specific training programs. These interconnected barriers can create a repeated pattern; inadequately trained residents may not want to serve as mentors, perpetuating educational gaps across generations in the absence of targeted intervention. To improve the training experience, participants expressed a clear preference for active, experiential learning, highlighting clinical exposure through structured rotations and mentorship as the most valuable methods for developing competence.

The challenges identified in our study are not unique to Canada and are mirrored by similar findings in other high-income countries, which have led to the development of innovative models of care. In Australia, AYA cancer programs such as the Victorian Adolescent and Young Adult Cancer Service have developed multidisciplinary models that integrate medical and psychosocial care, centralize complex care, and facilitate transition to survivorship [22]. Similarly, the United Kingdom has established a national network of Teenage and Young Adult (TYA) Cancer Services, with specialized Principal Treatment Centers (TYA PTCs) and a strong emphasis on holistic and psychosocial support [23]. These international examples provide valuable frameworks for Canada to develop a national strategy for AYA palliative care.

Our findings emphasize the need to move beyond passive, didactic lectures and create structured opportunities for hands-on clinical experience in AYA palliative care. The RCPFC s Area of Focused Competence in AYA Oncology demonstrates how competency-based curricula can be nationally implemented [10]. A similar approach for AYA palliative care will require coordinated action from the RCPSC, CFPC, and program directors to establish standards and allocate training resources.

### Limitations

While this study benefits from its national scope and the use of a bilingual questionnaire developed by a multidisciplinary expert committee with a patient partner, some limitations should be noted. The use of electronic surveys may introduce selection bias, as individuals with a stronger interest in AYA palliative care may have been more likely to participate, and non-responders or excluded participants may differ systematically from those included. The majority of participants trained at Ontario institutions, which may limit generalizability across Canadian regions. The data are based on self-reported comfort and knowledge, which may not accurately correlate with objective competence. The survey was not informed by a formal conceptual model of developmental considerations, and cognitive interviews with the study participants were not conducted during the survey development process. The format of some survey items might not have fully captured the participants’ educational needs or optimally encouraged detailed narrative responses. Finally, as the survey included graduates from up to six years prior, recall bias may have influenced responses regarding training experiences.

## Conclusion

This study highlights some gaps in the training of Canadian adult palliative care graduates in AYA care. The identified deficits in comfort and knowledge, particularly related to providing care to younger AYAs, psychosocial care, and process of care transitions, underscore an important opportunity for improvement within Canadian medical education. These findings may inform the development of AYA-specific competencies and future curriculum initiatives. The ultimate goal is to ensure that future palliative care specialists are better equipped with the skills and confidence to provide high-quality, compassionate care to this unique and vulnerable population.

## Supplementary Information


Supplementary Material 1.



Supplementary Material 2.


## Data Availability

The datasets used and/or analysed during the current study are available from the corresponding author on reasonable request.
