# Supplementary material for: Advancing palliative medicine training in adolescent and young adult care: a needs assessment study
Source: BMC Med Educ. 2026 Jun 22;26:1410. doi: 10.1186/s12909-026-09774-8 (PMC13536818; doi:10.1186/s12909-026-09774-8)
Supplement: Supplementary file 1 [file 12909_2026_9774_MOESM1_ESM.docx]

**Assessing the Educational Needs of Palliative Medicine Learners in Providing Care to Adolescents and Young Adults: A National Survey**

**Section 1: Demographics and Training Background**

1. **What is your current age?**

Choose one of the following answers

- Less than 30 years old
- 30-35 years old
- 36-40 years old
- 41-45 years old
- 46-50 years old
- More than 50 years old

2. **What is your sex?**

Choose one of the following answers

- Female
- Male
- Prefer not to say
- Other

3. **What is your current practice setting?**

 Check all that apply

- Academic hospital
- Community hospital
- Hospice care
- Home-based
- Outpatient
- Other

4. **Which of the following best describes your residency training background?**

Choose one of the following answers

- Royal College-accredited Palliative Care Program
- Family Medicine Palliative Care Program
- Other

5. **In which year did you complete your palliative care residency?**

Choose one of the following answers

- 2019
- 2020
- 2021
- 2022
- 2023
- 2024

6. **At which University did you complete your palliative care residency?**

Choose one of the following answers

- University of BC
- University of Calgary
- University of Alberta
- University of Manitoba
- Universite de Montreal
- McGill University
- McMaster University
- Universite de Sherbrooke
- University Laval
- Dalhousie University
- Western University
- University of Toronto
- Queen’s University
- University of Ottawa
- Other

7. **During your residency, how often did you care for AYA (Adolescents and Young Adults, aged 15-39 years) patients?**

Choose one of the following answers

- Never
- Once every few months
- At least once monthly
- Once or more per week
- Daily

**8. In your current practice, how often do you care for AYA patients?**

Choose one of the following answers

- Never
- Once every few months
- At least once monthly
- Once or more per week
- Daily

**Section 2: Exposure and Experience with AYA Palliative Care**

**9. How comfortable do you feel providing palliative care to patients in the following age groups?** (Not at all comfortable, Slightly comfortable, Moderately comfortable, Very comfortable, Extremely comfortable)

- 15-18 years
- 19-24 years
- 25-29 years
- 30-34 years
- 35-39 years

**10. Have you received any training specifically related to palliative care for AYA patients during your residency?**

- Yes
- No

**11. If yes, please specify the type of training received:**
Check all that apply

- Lectures or classroom-based learning
- Workshops or simulations
- Adult palliative care clinical rotations
- Pediatric palliative care rotation
- Online modules or e-learning
- Other

**12. Please rate your knowledge level in the following aspects of providing palliative care to AYA patients**
(Very Poor, Poor, Fair, Good, Very Good)

1. Pain management

2. Management of respiratory symptoms (e.g. dyspnea, cough)

3. Management of gastro-intestinal symptoms (e.g. nausea, vomiting, constipation, diarrhea)

4. Management of psychological symptoms (e.g. anxiety, depression, existential distress)

5. Navigating Social support resources

6. Exploring sexual health and fertility concerns

7. Discussing spiritual care needs

8. Discussing financial hardships

9. Process of care transition from pediatric to adult care

10. Goals of care discussions (e.g. active treatment preferences)

11. Managing complex ethical issues (e.g. patient’s autonomy and decision making)

12. Transition of location of care discussions (e.g. discharge plans after hospital admission)

13. Discussing end-of-life care with patients and their caregivers

14. Providing end-of-life care

15. Providing caregiver support

16. Grief and bereavement support to caregivers

17. Grief and bereavement resources for patients’ children

18. Providing home palliative care to AYA patients

19. Providing palliative care to AYAs in outpatient clinics

20. Providing palliative care to AYAs in inpatient settings

**13. Do you feel comfortable providing the following aspects of palliative care to AYA patients**?
(Strongly disagree, Disagree, Neutral, Agree, Strongly agree)

1. Explaining what palliative care means

2. Pain management

3. Management of respiratory symptoms (e.g. dyspnea, cough)

4. Management of gastro-intestinal symptoms (e.g. nausea, vomiting, constipation, diarrhea)

5. Management of psychological symptoms (e.g. anxiety, depression, existential distress)

6. Navigating Social support resources

7. Exploring sexual health and fertility concerns

8. Discussing spiritual care needs

9. Discussing financial hardships

10. Process of care transition from pediatric to adult care

11. Goals of care discussions (e.g. active treatment preferences)

12. Managing complex ethical issues (e.g. patient’s autonomy and decision making)

13. Transition of location of care discussions (e.g. discharge plans after hospital admission)

14. Discussing end-of-life care with patients and their caregivers

15. Providing end-of-life care

16. Providing caregiver support

17. Grief and bereavement support to caregivers

18. Grief and bereavement resources for patients’ children

19. Providing home palliative care to AYA patients

20. Providing palliative care to AYAs in outpatient clinics

21. Providing palliative care to AYAs in inpatient settings

**Section 3: Educational Needs and Gaps**

**14. During your palliative medicine residency training, how challenging were the following aspects of providing palliative care to AYA patients?**
(Not challenging at all, Slightly challenging, Moderately challenging, Very challenging, Extremely challenging)

- Limited knowledge of AYA-specific needs
- Lack of clinical exposure to AYAs during training program
- Time constraints during patient care
- Difficulty in addressing psychosocial concerns
- Emotional burden of working with younger patients
- Lack of AYA-specific resources at the site of the training program
- Limited interdisciplinary collaboration for AYA cases

**15. Have you encountered any other challenges while providing palliative care to AYA patients during your residency training?**

**16. To what extent do you believe the following areas require more formal training within palliative medicine residency programs?**

(Not at all needed, Slightly needed, Moderately needed, Very needed, Essential)

- Symptom management for AYA patients
- Pharmacology of the medications used for AYAs
- Communicating bad news and end-of-life discussions with AYAs
- Grief and bereavement support for AYA patients and their families
- Understanding developmental, psychological, and social factors unique to AYA patients
- Engaging caregivers in shared decision-making for AYA patients
- Process of care transition from pediatric to Adult medical care
- Providing inpatient palliative care for AYAs
- Home-based palliative care for AYAs
- Outpatient palliative care clinics for AYAs

**17. Can you identify any other areas you believe requires more formal training in palliative medicine residency programs for AYA care?**

**18. Which of the following patient age groups do you think require more focused training within the palliative medicine residency programs?**

Check all that apply

- 15-18 years
- 19-24 years
- 25-29 years
- 30-34 years
- 35-39 years
- None of the above
- All of the above

**Section 4: Barriers to Accessing Training**

**19. What barriers have you faced in obtaining training specific to AYA palliative care during your residency training?**
Check all that apply

- Lack of available AYA-specific training programs
- Insufficient allocated time within the residency schedule
- Lack of mentors or role models experienced in AYA care
- Limited clinical exposure to AYA patients
- Institutional focus on other patient populations
- Other

**Section 5: Preferred Learning Methods**

**20. Which learning formats would you find most useful for improving the learners’ skills in AYA palliative care?**
(Not at all useful, Slightly useful, Moderately useful, Very useful, Most useful)

- Online e-learning modules
- In-person workshops
- Mentorship
- Self-learning through reading literature
- Group discussions or peer support groups
- Case-based learning
- Clinical exposure through structured rotations

**21. Are there any other learning formats you would find useful?**

**Section 6: Final Comments**

**22. Do you have any additional comments or suggestions regarding AYA palliative care training?**

**23. If you would like to receive the compensation of a $50 Tim Horton’s gift card for completing the survey, please proceed to the following link to enter your name and e-mail address. Your contact information will not be linked to your responses.**

**[Insert link to Contact Information Survey]**

Thank you for your participation!

**Enquête nationale sur les besoins éducatifs des apprenantes et apprenants en médecine palliative dans la prestation de soins aux adolescents et aux jeunes adultes.**

**Partie 1 : Données démographiques et formation**

1. **Quel âge avez-vous ?**

Choisissez une seule réponse.

- Moins de 30 ans
- Entre 30 et 35 ans
- Entre 36 et 40 ans
- Entre 41 et 45 ans
- Entre 46 et 50 ans
- Plus de 50 ans

2. **Quel est votre sexe ?**

Choisissez une seule réponse.

- Féminin
- Masculin
- Je préfère ne pas répondre
- Autre sexe

3. **Quel est votre cadre d’exercice actuel ?**

 Cochez toutes les réponses pertinentes.

- Hôpital universitaire
- Hôpital communautaire
- Soins palliatifs
- À domicile
- Ambulatoire
- Autre cadre

4. **Lequel des énoncés suivants décrit le mieux votre formation de résident(e) ?**

Choisissez une seule réponse.

- Programme de soins palliatifs accrédité par le Collège royal
- Programme de soins palliatifs en médecine familiale
- Autre formation

5. **En quelle année avez-vous terminé votre résidence en soins palliatifs ?**

Choisissez une seule réponse.

- 2019
- 2020
- 2021
- 2022
- 2023
- 2024

6. **À quelle université avez-vous effectué votre résidence en soins palliatifs ?**

Choisissez une seule réponse.

- Université de la Colombie-Britannique
- Université de Calgary
- Université de l’Alberta
- Université du Manitoba
- Université de Montréal
- Université McGill
- Université McMaster
- Université de Sherbrooke
- Université Laval
- Université Dalhousie
- Université Western
- Université de Toronto
- Université Queen’s
- Université d’Ottawa
- Autre université

7. **Pendant votre résidence, à quelle fréquence avez-vous pris soin de patientes et patients AJA (adolescents et jeunes adultes, de 15 à 39 ans) ?**

Choisissez une seule réponse.

- Jamais
- Une fois tous les quelques mois
- Au moins une fois par mois
- Une fois ou plus par semaine
- Chaque jour

**8. Dans votre pratique actuelle, à quelle fréquence prenez-vous soin de patientes et patients AJA ?**

Choisissez une seule réponse.

- Jamais
- Une fois tous les quelques mois
- Au moins une fois par mois
- Une fois ou plus par semaine
- Chaque jour

**Partie 2 : Soins palliatifs aux AJA : exposition et expérience**

**9. Dans quelle mesure vous sentez-vous à l’aise de prodiguer des soins palliatifs aux groupes d’âge suivants ?** (Pas du tout, un peu, modérément, très, parfaitement)

- 15-18 ans
- 19-24 ans
- 25-29 ans
- 30-34 ans
- 35-39 ans

**10. Au cours de votre résidence, avez-vous reçu une formation spécifique en soins palliatifs aux AJA ?**

- Oui
- Non

**11. Si vous avez répondu oui, veuillez préciser le type de formation reçue.**
Cochez toutes les réponses pertinentes.

- Cours magistraux ou apprentissage en classe
- Ateliers ou simulations
- Stages cliniques en soins palliatifs pour adultes
- Stage en soins palliatifs pédiatriques
- Modules ou apprentissage en ligne
- Autre type de formation

**12. Veuillez évaluer votre niveau de connaissances dans les facettes suivantes de la prestation de soins palliatifs aux AJA**
(Très faible, faible, passable, élevé, très élevé)

1. Gestion de la douleur

2. Gestion des symptômes respiratoires (ex. : dyspnée, toux)

3. Gestion des symptômes gastro-intestinaux (par exemple, nausées, vomissements, constipation, diarrhée)

4. Gestion des symptômes psychologiques (par exemple, anxiété, dépression, détresse existentielle)

5. Naviguer dans les ressources et soutiens sociaux

6. Explorer les problèmes de santé sexuelle et de fertilité

7. Discuter des besoins en matière de soins spirituels

8. Discuter des difficultés financières

9. Processus de transition des soins pédiatriques aux soins pour adultes

10. Discussions sur les objectifs de soins (par exemple, préférences en matière de traitement actif)

11. Gestion de questions éthiques complexes (par exemple, l’autonomie du patient et la prise de décision)

12. Discussions sur la transition du lieu de soins (par exemple, plan de sortie après un séjour à l’hôpital)

13. Discuter des soins de fin de vie avec les patient(e)s et leurs aidant(e)s

14. Prodiguer des soins de fin de vie

15. Apporter du soutien aux personnes aidantes

16. Soutien aux personnes qui vivent de la tristesse ou un deuil

17. Ressources sur le chagrin et le deuil pour les enfants des patient(e)s

18. Soins palliatifs à domicile aux AJA

19. Soins palliatifs aux AJA en consultation externe

20. Soins palliatifs aux AJA en milieu hospitalier

**13. Vous sentez-vous à l’aise de prodiguer les facettes suivantes des soins palliatifs aux AJA ?**
(Pas du tout, un peu, moyennement, très, parfaitement)

1. Expliquer ce que sont les soins palliatifs

2. Gestion de la douleur

3. Gestion des symptômes respiratoires (ex. : dyspnée, toux)

4. Gestion des symptômes gastro-intestinaux (par exemple, nausées, vomissements, constipation, diarrhée)

5. Gestion des symptômes psychologiques (par exemple, anxiété, dépression, détresse existentielle)

6. Naviguer dans les ressources et soutiens sociaux

7. Explorer les problèmes de santé sexuelle et de fertilité

8. Discuter des besoins en matière de soins spirituels

9. Discuter des difficultés financières

10. Processus de transition des soins pédiatriques aux soins pour adultes

11. Discussions sur les objectifs de soins (par exemple, préférences en matière de traitement actif)

12. Gestion de questions éthiques complexes (par exemple, l’autonomie du patient et la prise de décision)

13. Discussions sur la transition du lieu de soins (par exemple, plan de sortie après un séjour à l’hôpital)

14. Discuter des soins de fin de vie avec les patient(e)s et leurs soignant(e)s

15. Prodiguer des soins de fin de vie

16. Apporter du soutien aux personnes aidantes

17. Soutien aux personnes qui vivent de la tristesse ou un deuil

18. Ressources sur le chagrin et le deuil pour les enfants des patient(e)s

19. Soins palliatifs à domicile aux AJA

20. Soins palliatifs aux AJA en consultation externe

21. Soins palliatifs aux AJA en milieu hospitalier

**Partie 3 : Besoins et lacunes en matière d’éducation**

**14. Au cours de votre résidence en médecine palliative, dans quelle mesure les facettes suivantes de la prestation de soins palliatifs aux AJA ont-elles été difficiles ?**
(Pas du tout, légèrement, moyennement, très, extrêmement)

- Connaissance limitée des besoins spécifiques des AJA
- Manque d’exposition clinique aux AJA pendant la formation
- Contraintes de temps pendant les soins aux patients
- Difficulté à répondre aux préoccupations psychosociales
- Le fardeau émotionnel du travail auprès de patient(e)s plus jeunes
- Manque de ressources spécifiques aux AJA là où j’ai fait ma résidence
- Collaboration interdisciplinaire limitée dans les cas d’AJA

**15. Avez-vous rencontré d’autres difficultés en prodiguant des soins palliatifs aux AJA au cours de votre résidence ?**

**16. Dans quelle mesure pensez-vous que les domaines suivants nécessitent une formation plus officielle dans le cadre des programmes de résidence en médecine palliative ?**

(Pas du tout, légèrement, modérément, très, absolument)

- Gestion des symptômes chez les AJA
- Pharmacologie des médicaments utilisés pour les AJA
- Communication des mauvaises nouvelles et discussions de fin de vie avec les AJA
- Accompagnement du chagrin et du deuil des patient(e)s AJA et de leurs proches
- Compréhension des facteurs développementaux, psychologiques et sociaux propres aux AJA
- Implication des personnes aidantes dans la prise de décision partagée des patient(e)s AJA
- Processus de transition des soins pédiatriques aux soins pour adultes
- Soins palliatifs aux AJA en milieu hospitalier
- Soins palliatifs aux AJA à domicile
- Soins palliatifs aux AJA en consultation externe

**17. Y a-t-il d’autres facettes des soins aux AJA qui, selon vous, nécessitent une formation plus officielle dans le cadre de la résidence en médecine palliative ?**

**18. Selon vous, quels groupes d’âge de patientes et patients parmi les suivants nécessitent une formation plus ciblée dans le cadre des programmes de résidence en médecine palliative ?**

Cochez toutes les réponses pertinentes.

- 15-18 ans
- 19-24 ans
- 25-29 ans
- 30-34 ans
- 35-39 ans
- Aucune de ces réponses
- Toutes ces réponses

**Partie 4 : Obstacles à l’accès à la formation**

**19. Au cours de votre résidence, quels obstacles avez-vous rencontrés dans l’obtention d’une formation spécifique aux soins palliatifs pour les AJA ?**
Cochez toutes les réponses pertinentes.

- Manque de programmes de formation spécifiques aux AJA
- Insuffisance du temps alloué dans le programme de résidence
- Manque de mentors ou de modèles chevronnés dans les soins aux AJA
- Exposition clinique limitée aux AJA
- L’institution met l’accent sur d’autres populations de patients
- Autre obstacle

**Partie 5 : Méthodes d’apprentissage préférées**

**20. À quel point les méthodes d’apprentissage suivantes sont-elles utiles à l’amélioration des compétences des personnes apprenantes en soins palliatifs aux AJA ?**
(Pas du tout, un peu, moyennement, très, extrêmement)

- Modules d’apprentissage en ligne
- Ateliers en personne
- Mentorat
- Autoapprentissage par la lecture de la documentation
- Discussions de groupe ou groupes de soutien par les pairs
- Apprentissage basé sur des cas concrets
- Exposition clinique par le biais de stages structurés

**21. Existe-t-il d’autres méthodes d’apprentissage que vous trouveriez utiles ?**

**Partie 6 : Derniers commentaires**

**22. Avez-vous d’autres commentaires ou suggestions à formuler concernant la formation en soins palliatifs aux AJA ?**

**23. Si vous souhaitez recevoir une compensation sous forme d’une carte-cadeau Tim Hortons de 50 $, le lien suivant vous permettra de nous transmettre votre nom et votre adresse électronique. Ces informations ne seront pas rattachées à vos réponses.**

**[Insérer le lien vers le questionnaire sur les coordonnées]**

Merci d’avoir répondu à l’enquête !
