# Supplementary material for: Advancing palliative medicine training in adolescent and young adult care: a needs assessment study
Source: BMC Med Educ. 2026 Jun 22;26:1410. doi: 10.1186/s12909-026-09774-8 (PMC13536818; doi:10.1186/s12909-026-09774-8)
Supplement: Supplementary file 2 [file 12909_2026_9774_MOESM2_ESM.docx]

**Table 1. Self-Rated Knowledge Levels Across AYA Palliative Care Competency Areas**

| **Competency Area** | **Knowledge level, n (%)^*^** | | |
| --- | --- | --- | --- |
|  | **High**  **(Good/Very good)** | **Moderate**  **(Fair)** | **Low**  **(Poor/Very poor)** |
| Pain management | 44 (84.6) | 6 (11.5) | 2 (3.8) |
| Management of respiratory symptoms | 45 (84.9) | 6 (11.3) | 2 (3.8) |
| Management of gastro-intestinal symptoms | 46 (86.8) | 6 (11.3) | 1 (1.9) |
| Management of psychological symptoms | 22 (43.1) | 19 (37.3) | 10 (19.6) |
| Navigating social support resources | 14 (26.9) | 18 (34.6) | 20 (38.5) |
| Exploring sexual health and fertility concerns | 6 (12) | 13 (26) | 31 (62) |
| Discussing spiritual care needs | 27 (55.1) | 15 (30.6) | 7 (14.3) |
| Discussing financial hardships | 18 (35.3) | 15 (29.4) | 18 (35.3) |
| Process of care transition from pediatric to adult care | 7 (13.7) | 9 (17.6) | 35 (68.6) |
| Goals of care discussions | 27 (52.9) | 19 (37.3) | 5 (9.8) |
| Managing complex ethical issues (e.g. patient autonomy and decision making) | 22 (44.9) | 15 (30.6) | 12 (24.5) |
| Transition of location of care discussions (e.g. discharge plans after hospital admission) | 36 (72) | 10 (20) | 4 (8) |
| Discussing end-of-life care with patients and caregivers | 33 (67.3) | 13 (26.5) | 3 (6.1) |
| Providing end-of-life care | 40 (80) | 9 (18) | 1 (2) |
| Providing caregiver support | 34 (69.4) | 10 (20.4) | 5 (10.2) |
| Grief and bereavement support to caregivers | 25 (50) | 16 (32) | 9 (18) |
| Grief and bereavement resources for patients' children | 16 (32) | 21 (42) | 13 (26) |
| Providing home palliative care to AYA patients | 18 (36) | 17 (34) | 15 (30) |
| Providing palliative care to AYAs in outpatient clinics | 26 (52) | 11 (22) | 13 (26) |
| Providing palliative care to AYAs in inpatient settings | 31 (62) | 11 (22) | 8 (16) |

^*^ Percentages are based on the total number of eligible answers in each question. Item-specific denominator ranging from n=49 to n=53 due to missing data

**Table 2. Comfort Levels Across AYA Palliative Care Competency Areas**

| **Competency Area** | **Comfort level, n (%)^*^** | | |
| --- | --- | --- | --- |
|  | **High**  **(Agree/Strongly agree)** | **Moderate**  **(Neutral)** | **Low**  **(Disagree/Strongly disagree)** |
| Explaining what palliative care means | 48 (92.3) | 1 (1.9) | 3 (5.8) |
| Pain management | 48 (94.1) | 1 (2) | 2 (3.9) |
| Management of respiratory symptoms | 49 (96.1) | 1 (2) | 1 (2) |
| Management of gastro-intestinal symptoms | 49 (96.1) | 1 (2) | 1 (2) |
| Management of psychological symptoms | 35 (68.6) | 11 (21.6) | 5 (9.8) |
| Navigating social support resources | 22 (44) | 14 (28) | 14 (28) |
| Exploring sexual health and fertility concerns | 9 (18) | 13 (26) | 28 (56) |
| Discussing spiritual care needs | 30 (60) | 13 (26) | 7 (14) |
| Discussing financial hardships | 24 (48) | 11 (22) | 15 (30) |
| Process of care transition from pediatric to adult care | 8 (16.3) | 13 (26.5) | 28 (57.1) |
| Goals of care discussions | 37 (74) | 10 (20) | 3 (6) |
| Managing complex ethical issues (e.g. patient autonomy and decision making) | 26 (52) | 14 (28) | 10 (20) |
| Transition of location of care discussions (e.g. discharge plans after hospital admission) | 36 (72) | 10 (20) | 4 (8) |
| Discussing end-of-life care with patients and caregivers | 39 (78) | 8 (16) | 3 (6) |
| Providing end-of-life care | 42 (84) | 6 (12) | 2 (4) |
| Providing caregiver support | 38 (76) | 9 (18) | 3 (6) |
| Grief and bereavement support to caregivers | 29 (58) | 15 (30) | 6 (12) |
| Grief and bereavement resources for patients' children | 19 (38) | 21 (42) | 10 (20) |
| Providing home palliative care to AYA patients | 25 (51) | 12 (24.5) | 12 (24.5) |
| Providing palliative care to AYAs in outpatient clinics | 29 (58) | 11 (22) | 10 (20) |
| Providing palliative care to AYAs in inpatient settings | 38 (76) | 6 (12) | 6 (12) |

^*^ Percentages are based on the total number of eligible answers in each question. Item-specific denominator ranging from n=49 to n=52 due to missing data
